# Supplementary material for: Mechanisms governing the pioneering and redistribution capabilities of the non-classical pioneer PU.1
Source: Nat Commun. 2020 Jan 21;11:402. doi: 10.1038/s41467-019-13960-2 (PMC6972792; doi:10.1038/s41467-019-13960-2)
Supplement: Supplementary file 7 — Source data [file 41467_2019_13960_MOESM7_ESM.zip › Source_Data/FigureS3/FigureS3J_MotifScanOutput/homerResults/motif12.similar.html]

motif12

## Information for motif12

C
T
A
G
G
C
A
T
C
A
G
T
C
A
G
T
C
G
T
A
G
T
A
C
C
G
T
A
A
G
T
C
C
T
G
A
T
C
G
A
C
G
T
A
T
C
A
G
T
A
G
C
  
Reverse Opposite:  

A
T
C
G
A
G
T
C
G
C
A
T
A
G
C
T
A
G
C
T
C
T
A
G
G
C
A
T
C
A
T
G
G
C
A
T
G
T
C
A
G
T
C
A
C
G
T
A
G
A
T
C
  

|  |  |
| --- | --- |
| p-value: | 1e-113 |
| log p-value: | -2.609e+02 |
| Information Content per bp: | 1.568 |
| Number of Target Sequences with motif | 4525.0 |
| Percentage of Target Sequences with motif | 34.29% |
| Number of Background Sequences with motif | 8738.2 |
| Percentage of Background Sequences with motif | 23.94% |
| Average Position of motif in Targets | 393.7 +/- 341.8bp |
| Average Position of motif in Background | 319.1 +/- 202.4bp |
| Strand Bias (log2 ratio + to - strand density) | -0.1 |
| Multiplicity (# of sites on avg that occur together) | 1.21 |
| Motif File: | file (matrix) reverse opposite |

### Similar de novo motifs found

|  |  |  |  |  |  |  |  |
| --- | --- | --- | --- | --- | --- | --- | --- |
| Rank | Match Score | Redundant Motif | P-value | log P-value | % of Targets | % of Background | Motif file |
| 1 | 0.782 | C A G T A C G T C T G A G T C A G C A T A G T C G C T A | 1e-103 | -239.121527 | 37.18% | 26.98% | motif file (matrix) |
| 2 | 0.764 | G A T C G C A T T C A G C G A T C T A G A G C T C G T A G T A C G C T A | 1e-55 | -127.310538 | 17.22% | 11.69% | motif file (matrix) |
| 3 | 0.793 | C T A G G A C T T G C A A G T C C T G A A G T C C G T A C T A G | 1e-44 | -102.803736 | 28.26% | 22.10% | motif file (matrix) |
| 4 | 0.793 | T A C G C A G T A C G T A C G T A C T G A G C T C A T G A G C T T C G A G T C A A T C G A G C T C T G A A G T C | 1e-32 | -74.029498 | 0.70% | 0.07% | motif file (matrix) |
